# Shape-Selective Assembly of Anisotropic, Deformable Microcomponents Using Bottom-Up Micromanufacturing

**DOI:** 10.3390/mi7040068

**Published:** 2016-04-14

**Authors:** Gunjan Agarwal, Carol Livermore

**Affiliations:** 1 Department of Mechanical Engineering, École Polytechnique Fédérale De Lausanne (Swiss Federal Institute of Technology), 1003 Lausanne, Switzerland; 2 Department of Mechanical and Industrial Engineering, Northeastern University, Boston, MA 02115, USA

**Keywords:** self-assembly, micromanufacturing, sorting, anisotropic, deformable, hydrogel, stop flow lithography

## Abstract

A technique for shape-selective directed assembly of anisotropic, deformable, chemically-identical microcomponents onto patterned rigid templates based on shape and size differences is modeled and demonstrated. The assembly method not only controls the selective placement of the components, but also aligns the components with the assembly sites. Unlike the assembly of isotropic (spherical) microcomponents, in which only size differences can be used to discriminate among chemically-identical components to achieve selective placement, differences in both shape and size can enable selectivity in the assembly of anisotropic (non-spherical) microcomponents. The present selective directed assembly is driven by shape-matching to a microfabricated template to provide selectivity, uniform chemical surface functionalization to promote assembly, and megasonic excitation to prevent assembly into poorly shape-matched binding sites. A theoretical framework quantifies the predicted selectivity of this approach and predicts that it will be effective for many material combinations, including hydrogels and bio-compatible polymers. Experiments demonstrate successful directed assembly of cylindrical, hydrogel colloidal microcomponents with 26 μm mean diameter and 50 μm length into silicon templates patterned with hemicylindrical assembly sites. During the assembly, tapered microcomponents with 150 μm length and a nominal diameter of 26 μm that decreases along the components’ lengths were successfully excluded from hemicylindrical assembly sites. These results provide the first demonstration of selective directed assembly of non-spherical microcomponents by this approach. The assembly shows high local yields in agreement with theory.

## 1. Introduction

The requirements for directed assembly of microcomponents [1,2] depend on what is being assembled. In [3,4,5,6,7,8,9], for example, directed assembly is used to locate microspheres in their intended locations [3,4,5,6] and groupings [7,8,9]. In [10,11,12,13,14,15,16], directed assembly is used to locate anisotropic (non-circular) microplates in their intended locations and orientations to ensure functioning of the system. Assembly of anisotropic components with the correct orientation requires that an increased degree of control be engineered into the energy landscape of the directed assembly system [17].

The simultaneous, selective assembly of microspheres and nanospheres of different diameters from liquid into size-matched hemispherical wells in a patterned template has been demonstrated previously [18,19,20,21,22,23]. In this approach, a spatially uniform surface attraction between the spheres and the surface of the template drives adhesion of the spheres onto the surface. The reduction in system energy is greatest when a sphere adheres in a well that matches the sphere’s diameter, so that the area over which the energy is reduced is maximized. Megasonic excitation (*i.e.*, acoustic excitation in the MHz frequency range) applied to the liquid creates fluid forces that roll loosely-bound, incorrectly-assembled microspheres out of their assembly sites (wells) while leaving tightly-bound, correctly-assembled microspheres in their assembly sites. Since rolling modifies the contact area (the area over which surface energy is reduced) gradually as compared with the abrupt change in contact area that would occur during direct lift-off of a microsphere from an assembly site, a relatively modest and spatially uniform megasonic excitation is sufficient to remove incorrectly-assembled microspheres from mismatched assembly sites. By selectively removing only incorrectly-assembled elements, these effects enable the simultaneous, selective, directed assembly of microspheres and nanospheres into their intended assembly wells on a template surface [19].

**Figure 1:**
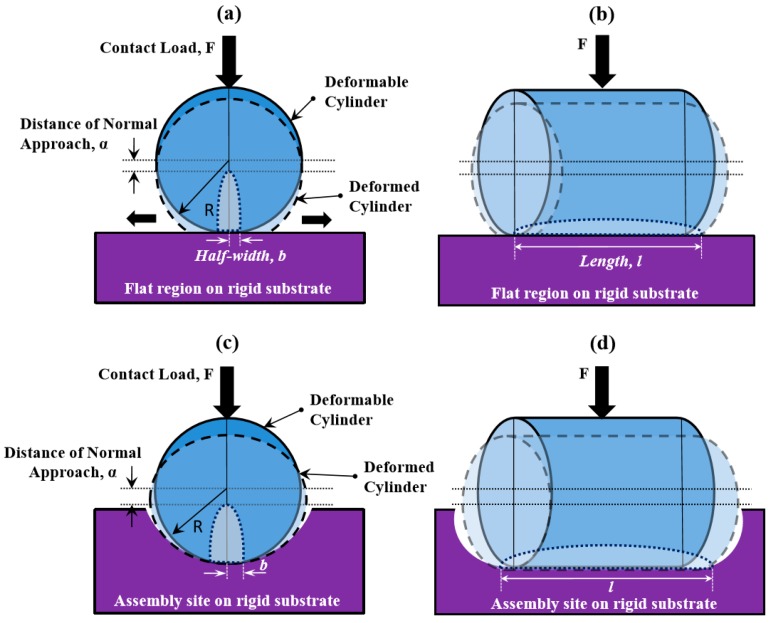
(**a**,**b**) Schematic diagrams of cylindrical components located within hemicylindrical wells that have rounded end caps shown in an axial view (a) and in a side view (b). (**c**,**d**) Schematic diagrams of cylindrical components located on a flat surface shown in an axial view (c) and in a side view (d). The original geometry is shown with solid outlines in darker blue, and the deformed geometry for the case of Hertzian contact under the influence of a contact load is shown with dotted outlines in lighter blue.

Anisotropic, non-spherical components offer the potential to achieve a new level of selectivity using the directed assembly processes of [18,19,20,21,22,23]. Namely, shape selectivity can combine with size selectivity to enable creation of systems with more diverse structures and applications (e.g. optical metamaterials, 3D microelectromechanical systems (MEMS) devices, or shape-driven cell sorting). This paper extends the models and experimental methods for the directed assembly of isotropic spherical components to anisotropic components, and in particular to the selective, directed assembly of cylinders into hemicylindrical assembly sites with rounded end caps. The cylindrical components and hemicylindrical assembly sites are shown schematically in Figure 1. The one-dimensional curvature of cylindrical microcomponents offers the potential for successful directed assembly via the rolling-based selective removal mechanism of [18,19,20,21,22,23]. More broadly, the rolling-based assembly methods of [18,19,20,21,22,23] can potentially be applied to other anisotropic components besides cylinders. As discussed in [18], however, this method is limited to the assembly of components with curvature in at least one direction because flat surfaces are not conducive to rolling.

Although cylinders’ curvature can potentially enable selective assembly by this method, the cylinders’ anisotropy presents two new challenges. The first is a weaker selective mechanism for assembly of cylinders than for assembly of spheres. The assembly mechanism is based on differences in contact area between correctly-assembled and incorrectly-assembled components. Since a sphere curves away from an underlying surface in two dimensions and a cylinder curves away from an underlying surface in only one dimension, the contact area selectivity will be weaker for cylinders than for spheres. The challenge of limited contact area selectivity for cylinders is exacerbated by the potential for deformations in the assembling components [20]. The second new challenge is that cylinders must be correctly oriented on the surface in order to even enter an assembly well. It is expected that components with less regular curvature will face still greater challenges in achieving correct orientation. The models and experiments presented here address both of these challenges for cylinders and enable the first demonstration of directed assembly of anisotropic, cylindrical, deformable components by the methods of [18,19,20,21,22,23].

## 2. Model of Cylinder Assembly

When rigid microspheres are assembled by this method, the success of the assembly process can be predicted by models [18,19,20] that describe how the contact area between the components and the template reduces the energy of the system. The energy varies as a sphere rolls from inside its assembly well to outside its assembly well, resulting in torques that oppose any effort to roll the sphere out of the well. The torque is calculated as the derivative of the energy with respect to the angular position of the sphere within the assembly well; this is enabled by the fact that the energy is solely a function of the sphere’s position within the well. If this torque is small enough, it can be overpowered by the fluidic forces from the megasonic excitation, which create torques that tend to roll the microspheres out of the wells. The balance between these torques determines whether the microsphere remains in the well or is removed from it.

The situation is similar for cylindrical components, in the sense that fluid forces create torques that tend to roll microcylinders out of hemicylindrical wells while surface interactions create torques that act to retain the microcylinders in the hemicylindrical wells. The change in geometry affects the equations for determining the contact area and energy in the system, since the case of contact between a sphere and flat surface (or between a sphere and a hollow hemispherical assembly site) involves contact about a point, whereas the case of contact between a cylinder and a plane (or between a cylinder and a hollow hemicylindrical site) involves contact about a line. The equations governing assembly of cylindrical microcomponents into hemicylindrical wells by this approach are presented below.

The selectivity of the assembly process is described in terms of the ratio of moments that promote component retention to moments that promote component removal. If the retention torque exceeds the removal torque, then the component is selectively retained inside the assembly site. If the removal torque is larger, then the mismatched component is successfully removed from the site. An exact analysis of selectivity for a given component and site requires the detailed shape of the assembly site and lacks generality. To show the structure of the selective removal model and illustrate trends in its operation, the model is instead presented using an idealized expression for the geometry of the assembly site.

The mechanical moment that acts to retain a component inside an assembly site depends on the interfacial energy γ between the assembly fluid and the surfaces and on the contact area between the cylindrical component and the hemicylindrical assembly site. The area $A_{n}$ of a cylindrical component that is nominally in contact with the assembly site is determined by identifying the points on the cylinder that are within a nominal adhesive distance *d_a_* of the surface of the assembly site. The actual contact area is always less than this nominal value because of surface roughness, which acts to increase the separation of parts of the surface beyond the adhesive distance. Roughness reduces the contact area by a factor of $C_{r}$ as compared with the nominal contact area, where $C_{r}$ is the coefficient of roughness of the surface. The retention moment is then calculated as

(1) $$M_{\text{retention}}=-\gamma C_{r}\frac{dA_{n}}{d\emptyset }$$

where $A_{n}$ is differentiated with respect to the rolling angle ∅ of the cylinder inside the assembly site. The angle ∅ is zero at the bottom of the assembly site and increases as the component travels up the walls of the site.

For the purposes of this idealized analysis, the radius of curvature of the hemicylindrical site is assumed to be a minimum $R_{\text{min}}$ at the bottom of the well and to increase linearly with ∅. This is qualitatively similar to the real case, in which nonideal mask adhesion during the microfabrication process produces assembly sites with a larger radius of curvature near the top of the well than near the bottom of the well. The idealized radius of curvature $R_{t}$ is then given as

(2) $$R_{t}\left(\emptyset \right)=\;R_{\text{min}}\left(1+\beta \emptyset \right)$$

where $R_{\text{min}}$ is also equal to the etched depth of the assembly site. The parameter β measures the rate of change of radius of curvature with angle ∅.

The cylindrical component defines a curved rectangular contact region as it rolls inside the hemicylindrical site. Its nominal contact area $A_{n}\;$is given by

(3) $$A_{n}=\;2\sqrt{2R_{eq}d_{a}}l_{c}$$

where $l_{c}$ is the length of the cylindrical component. $R_{eq}$ is the effective radius of curvature that takes into account both the radius of curvature of the site on the template ($R_{t}$) and the radius of the cylindrical component ($R_{c}$) and is described by

(4) $$\frac{1}{R_{eq}}=\frac{1}{R_{c}}+\frac{1}{R_{t}}$$

The removal moment arises from the primary (vertical) forces from the megasonic excitation acting on the center of mass of the cylindrical component. The component is preferentially located at the position within the assembly well where the contact area between component and assembly site is greatest. At this location, the component’s center of mass is laterally offset from the center of its contact with the well, so that the vertical forces and the horizontal lever arm produce a mechanical moment that tends to roll the cylindrical component from its well. Although the megasonic excitation produces a number of different primary forces [18,19], the dominant vertical force at this size scale is the added mass force,

(5) $$F_{am}=C_{am}\rho \pi R_{c}^{2}l_{c}\frac{dU}{dt}$$

where *C_am_* is the added mass factor (a constant of approximately 0.75 for the present component geometry), ρ is the density of the assembly fluid mixture, and *U* is the velocity of the cylinder under the fluid forces. The acceleration *dU/dt* is calculated as in [18,19], yielding a retention moment by the method of [19] as

(6) $$M_{\text{removal}}=\left(C_{am}\pi^{2}fR_{c}^{3}l_{c}\sqrt{\left(\frac{I\rho }{c}\right)}\right)\sin\theta$$

where *f* is the frequency of acoustic excitation, *I* is the intensity of the incident acoustic excitation, and *c* is the speed of sound in the assembly fluid. The angle θ is the non-zero angle formed between the vertical direction and the line joining the point of contact (between the cylinder and the site, as the cylinder rolls inside the site) and the center of the cylinder.

The selectivity is then determined by taking the ratio of the magnitude of the retention moment (1) to the removal moment (6). If the ratio is greater than one for a given geometry, then the component is expected to be retained in the site. If the ratio is less than one for a given geometry, then the component is expected to be removed from the site. For values of the ratio that are close to one, intermediate assembly yields (neither very high nor very low) are expected.

As an illustrative example, the model’s predictions are examined for a set of parameters that are similar though not identical to those of the present experiments. The assembly site’s maximum and minimum radii of curvature are taken to be 13.5 μm (at the template surface) and 10 μm (at the deepest central point in the site), corresponding to a value of 0.22 for the coefficient β. The interfacial energy γ is taken to be 8 mJ/m^2^, the coefficient of roughness *C_r_* is taken to be 0.03 (similar to previous experiments [18]), and the length of the component *l_c_* is taken to be 50 μm. The velocity *U* is calculated as in [18] to be

(7) $$U=\sqrt{\left(\frac{I}{\rho c}\right)}=4.3\text{ mm}/s$$

Using these parameter values and Equations (1) to (7), the ratios of maximum retention moment to maximum removal moment are calculated for several different radii of the cylindrical components (Table 1). The results illustrate the sensitivity of the selective assembly to variations in component geometry. For the best-matched components (a radius of 13.5 μm), the retention moment is higher than the removal moment, so that components are expected to be retained inside the sites. As the components’ dimensions decrease relative to the dimensions of the site, the ratio of retention moment to removal moment declines, so that components are expected to be removed from the sites by fluidic forces.

When deformable microspheres are assembled by the selective removal method, the situation is more complicated [21]. The surface interaction produces forces that attract the components to the surface. If the components are not rigid, the attractive forces cause the spheres to deform. Elastic deformation does not disrupt the assembly process; in equilibrium, the energy reduction due to additional surface contact is exactly balanced by the stored elastic energy. As described in [21], the fact that the energy is still solely a function of the sphere’s position within the assembly well for the case of elastic deformation ensures that the competing torques mechanism still works as for the case of rigid components. However, plastic deformation can disrupt the assembly process because some of the energy gained by increased surface contact area is dissipated. The tolerance for deformation of microspheres and/or their assembly templates was modeled in [21,23].

The detrimental effects of plastic deformation on the selective assembly of cylinders are conceptually similar to the effects of plastic deformation on assembly of spheres, but the mathematics changes with the geometry. The Hertzian expressions for mechanics of contact are used to determine the approach distance for a cylinder in contact with a flat substrate, or for a cylinder in contact with a substrate with a specified curvature, which in turn predicts the onset of plastic deformation. When a cylindrical component with radius *R_c_* is brought into contact with a cylindrical well having local radius of curvature *R_t_* (with the axes of the component and the template site being parallel) by the application of a force (a contact load) with magnitude *F*, the resulting contact region is an elliptical area with half-width *b* and length *l*, equal to the length of contact along the axes of the cylinders. Using the Hertzian contact theory [24], *b* is calculated to be

(8) $$b=\sqrt{\frac{4FR_{eq}}{\pi lE^{\prime }}}$$

The parameter *E′* is the combined modulus of the component and template materials, given by

(9) $$\frac{1}{E^{\prime }}=\frac{\left(1-\nu_{c}^{2}\right)}{E_{c}}+\frac{\left(1-\nu_{t}^{2}\right)}{E_{t}}$$

The parameters *E_c_* and *E_t_* are the moduli of the component and the template material, respectively, and *ν_c_* and *ν_t_* are the corresponding Poisson’s ratios for the two materials. The maximum contact pressure *p*_max_ between the component and the template under a force *F* is defined by

(10) $$p_{\text{max}}=\frac{2F}{\pi bl}={\left(\frac{FE^{\prime }}{\pi R_{eq}l}\right)}^{1/2}\;$$

and the mean pressure over the contact area is

(11) $$p_{avg}=\left(\frac{\pi }{4}\right)p_{\text{max}}$$

The axis of the cylinder moves closer to the surface by a distance of normal approach characterized by α [24] as

(12) $$\alpha =\frac{F}{\pi lE^{\prime }}\left[1+\text{ln}\left(\frac{4\pi l^{3}E^{\prime }}{FR_{eq}}\right)\right]$$

This value of normal approach is valid until the onset of plastic deformation. At initial yield, upon the onset of plastic deformation, the value of maximum pressure is given by [25]

(13) $$p_{\text{max}}=KH$$

where *H* is the hardness of the cylinder material and is roughly 3 times the yield strength *Y.* The component material’s hardness coefficient *K* is calculated in [26] and is given by the relation

(14) $$K=0.454+0.41\nu_{c}$$

Combining Equations (10) and (12)–(14), the critical approach distance at which plastic deformation begins in the cylinder is given by

(15) $$\alpha_{c}=\frac{K^{2}H^{2}R_{eq}}{{E^{\prime }}^{2}}\left[1+\text{ln}\left(\frac{4l^{2}{E^{\prime }}^{2}}{K^{2}H^{2}R_{eq}{}^{2}}\right)\right]$$

When *α/α_c_* is less than 1, the deformation is purely elastic, the cylindrical assembly model described by Equations (1)–(7) is predicted to be applicable, and assembly is expected to be successful. If the calculated ratio exceeds one, the model of Equations (1)–(7) will no longer be valid and the assembly may be only marginally successful, depending on the extent of deformation.

The mechanical behavior of the deformable components is described using an elastic-perfectly plastic model. The calculated *α/α_c_* ratios for a selection of component materials for the case of a 26-μm diameter, 50-μm long cylinder deforming on a flat silicon surface are given in Table 2. This case is illustrated in Figure 1c,d. For the purposes of Table 2, the contact force *F* is calculated as

(16) $$F=\frac{C_{r}A_{n}\gamma }{d_{a}}$$

Assuming a hydrophobic chemical attraction with an interfacial energy of 8 mJ/m^2^ as described above for the given geometry but with a roughness factor of 0.32, this yields a contact force of 3.4 × 10*^−^*^5^ N. The roughness factor is conservatively overestimated to exceed that of the present system by a factor of ten to ensure that predictions of successful assembly are not contingent upon having poor surface finish. The geometry and hydrophobic interaction strength reflect the experimental system used here.

Table 2 includes thermoplastic polymers such as polystyrene and PMMA, hydrogels such as poly(ethylene glycol) (PEG) and poly(lactic acid) (PLA), rubbery silicones such as PDMS, and other highly deformable biomaterials, including biological cells. Although the interactions and interfacial energies of these materials will vary with the details of the assembly system, a uniform interfacial energy of 8 mJ/m^2^ is assumed to better highlight the effects of their varying mechanical properties. The deformation thresholds for the onset of plastic deformation in the diverse materials of Table 2 vary by more than five orders of magnitude, but the corresponding values of *α/α_c_* are universally below the critical value of 1 for the case of interaction with a flat surface, indicating that mechanical contact will not disrupt the assembly process under these conditions. Ratios that are well below 1 reflect a greater margin of safety due to a favorable combination of mechanical properties (stiffness, compressibility and yield strength).

The effects of template geometry on deformation are shown in Table 3, which tabulates the *α/α_c_* ratio values for the materials of Table 2 for the case in which a cylindrical component is assembled in a hemicylindrical assembly well with a mean diameter of 27 μm, as in the case shown in Figure 1a,b. The contact force in this case is taken to be 3.3 × 10*^−^*^3^ N; the larger force reflects the greater contact area in a more shape-matched well. For all materials examined here, both the approach distance and the critical approach distance are larger for a cylindrical component in a hemicylindrical well than for a cylindrical component on a flat surface. The increase in approach distance reflects both the increased effective radius of curvature and the increased contact load (adhesion force), which result in an increased contact area between the component and the site as compared with contact on a flat surface. In most cases, including for hydrogels such as PEG, the approach distance increases more quickly than the critical approach distance, so that the *α/α_c_* ratio is larger for a component in a well than for a component on a flat surface. The exceptions are PDMS and materials with mechanical properties similar to those of biological cells, for which assembly in a well reduces the deformation ratio as compared with assembly on a flat surface. These results highlight the impact of mechanical properties on the advent of plastic deformation in the assembly system. In all cases, the deformation ratio remains below the critical value of 1 under these conditions, though polystyrene’s ratio of about 0.9 approaches the limit. The models show that typical hydrogel materials such as the one used in the present experiments are further from the threshold for permanent deformation than many other materials are. In addition, because the load-bearing area of the cylinders is greater than the load-bearing area of similarly-sized spheres, cylinders offer a greater resistance to the onset of plastic deformation than do spheres of the same material and approximate dimensions.

## 3. Experiments

### 3.1. Fabrication of Cylindrical Components and Limitations

The microcylinders were fabricated using stop flow lithography (SFL) [27,28,29]. They were prepared using a combination of 95% trimethylolpropane triacrylate (TMPTA) water-insoluble monomer and 5% photo-initiator (monomethyl ether hydroquinone), and they are naturally hydrophobic. A brief overview of the fabrication process is provided here, with more details described in [27,28,29]. The SFL technique synthesizes polymer particles from a flow driven incrementally through PDMS microchannels by compressed air. When the flowing stream of oligomer is stopped, an array of particles is polymerized from the flow using patterned UV light. The particles are then flushed out at high flow rates, and the cycle of stop-polymerize-flow is repeated.

The assembly process is sensitive to small variations in the size matching between components and assembly sites. To maintain dimensional uniformity, two sets of microcylinders are created, each in a single production run, and the cylinders are reused for multiple experiments. The first set of cylinders is 26 μm in diameter and 50 μm in length with a tight tolerance of ± 0.5 μm on the diameter distribution. A second set of cylinders is nominally 26 μm in diameter with a length of 150 μm. The diameters of the longer cylinders are non-uniform because of resolution limits of the SFL system, tapering significantly along the cylinders’ length as shown in the results section. Because their diameter varies along their length, the longer cylinders are poorly matched to the geometry of the assembly sites, which have uniform diameter along their length. By comparing assembly of the uniform-diameter components with assembly of the nonuniform-diameter components, assembly selectivity may be confirmed.

Although using a single production run ensures that the components are uniform in size, it imposes a limit on assembly yield. For cylinders of this diameter, the batch size is limited by throughput considerations to of order 10,000 components, on the order of ten times as many components as assembly sites. The assembly technique described here works best with an oversupply of components, with typically 10^3^–10^4^ times as many components as the number of assembly sites [21]. The relatively low numbers of components limits the average assembly yields that can be achieved, though selectivity can still be clearly seen through local assembly results.

### 3.2. Template Fabrication

For cylindrical microcomponents, size matching means diameter matching rather than length matching. To ensure diameter matching, the assembly wells are isotropically etched to the desired depth in an approximately 10 μm thick silicon dioxide layer on a silicon wafer. By etching from a mask pattern of narrow lines, approximately hemicylindrical wells are formed. The final wells are slightly wider than they are deep because of the finite width of the mask pattern and undercutting of the mask during etching. The wafer and the fabrication process include assembly wells with two different etch depths (and therefore two different widths) to enable matching to 8 μm diameter and 26 μm diameter cylinders. However, only the larger diameter wells are used in the present experiments because the 26 μm diameter cylinders offer tight dimensional uniformity for more conclusive results. Since the 8 μm wells are not used, the details of the additional fabrication process steps necessary to create them are omitted here; only the fabrication of the 26 μm wide wells is described.

**Figure 2:**
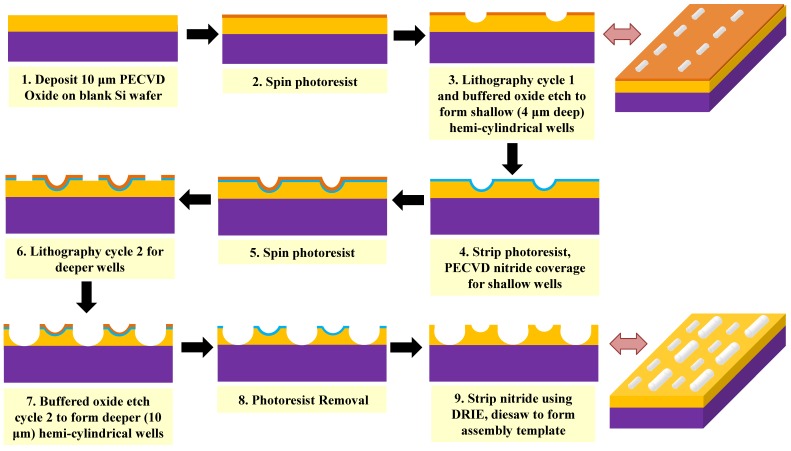
Schematic diagrams of the fabrication of the substrate. A plasma enhanced chemical vapour deposition (PECVD) silicon dioxide layer is deposited on a silicon substrate and patterned with photoresist. The oxide is isotropically etched with buffered oxide etch (BOE) through the patterned mask to form the first set of nearly hemicylindrical assembly wells. A layer of PECVD silicon nitride is deposited to protect the first set of wells during the subsequent processing and is patterned to expose the locations where the second set of wells will be created. The second set of features is lithographically patterned in a layer of photoresist, and wells are isotropically etched from the second set of features. The silicon nitride is removed, and the individual dies are separated.

The fabrication process is shown in Figure 2. An approximately 10 μm thick layer of silicon dioxide is deposited on a 150 mm diameter silicon wafer by plasma enhanced chemical vapour deposition (PECVD). After the shallower wells are patterned, a protective 0.5 μm thick layer of PECVD silicon nitride is deposited on the wafer and patterned with photolithography and deep reactive ion etching (DRIE) to expose the areas where the deeper wells are to be formed. The wafer is then spin-coated with 1.3 μm of OCG 825-20CS photoresist, and linear openings (1 μm wide and 80 μm, 120 μm and 200 μm long) are photolithographically patterned in the resist.

**Figure 3:**
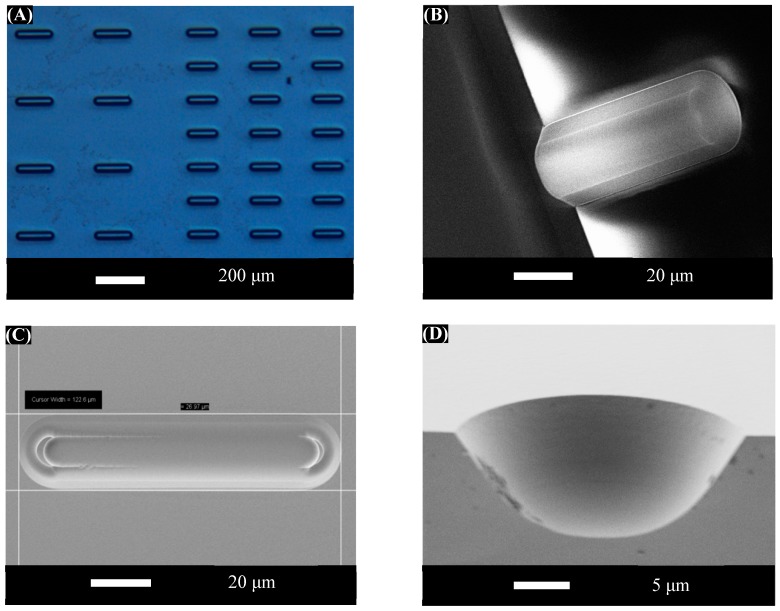
(**A**) Optical micrograph of a portion of the template showing assembly sites with diameter of 26 μm and two different lengths and spacings. (**B**) Angled scanning electron microscope (SEM) cross-sectional cutaway image of one of the assembly sites. (**C**) Top view SEM image of an assembly site. (**D**) Cross-sectional cutaway SEM image of an assembly site showing the smooth sidewalls created using isotropic BOE etching.

The exposed oxide is etched in buffered oxide etch (BOE) to a depth of 10 μm, with degas applied at the highest power for 5 s out of every 30 s of the total etch time, to form the deeper set of assembly wells. The frequent degassing procedure ensures that the etching process is not hindered by any air bubbles trapped within the deep cavities. The resulting wells are approximately 10 μm deep with a width of 27 μm, providing an acceptable shape match to the microcylinders. Finally, the remaining silicon nitride is removed by DRIE, and the wafer is diced. After a piranha clean and both water and ethanol rinses, the template is functionalized with a self-assembled monolayer of octadecyltrichlorosilane (OTS) to make it hydrophobic and promote component adhesion. A fabricated template is shown in Figure 3.

The assembly wells are organized into arrays with values of edge-to-edge spacing ranging between 50 and 300 μm. The various array designs are repeated across the surface of the 8 mm square die; this helps prevent spatial variations across the assembly system from being confused with the variation of yield due to differences in array design. This chip is designed with a total of 240 of the 26 μm diameter assembly sites on any single template. In addition, because the wells are in some cases several times longer than the microcylinders, it is possible for a given assembly well to accommodate several components arranged end-to-end in a line.

### 3.3. Assembly Procedure

**Figure 4:**
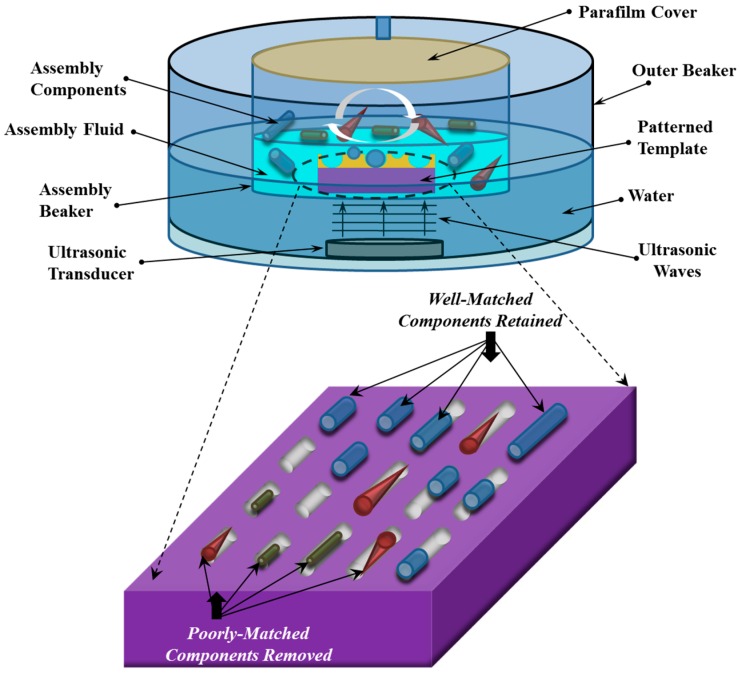
Conceptual diagram of the assembly set up (above), showing the assembly template and the microcomponents within the assembly beaker as well as the placement of the assembly beaker within an outer beaker that contains the high frequency ultrasonic transducer. The zoomed-in view (below) is a conceptual diagram of an assembly template during the assembly process. At the time shown, both well-matched and poorly-matched components have entered the assembly sites, and the poorly-matched components have not yet been removed by the selective removal mechanism.

The experimental approach and apparatus are shown in Figure 4 and are similar to those described in [18,19,20,21,22,23]. The assembly template and both types of microcylinders (the shorter, straight-walled ones and the longer, tapered ones) are placed together in the assembly fluid (8% water and 92% ethanol by volume) inside a small beaker that is suspended over a 1.7 MHz acoustic transducer (MMDIT-1.7, by Advanced Sonics, Oxford, CT, USA) in a larger, water-filled beaker. The transducer provides the acoustic excitation that enables the selectivity in the assembly process. The transducer’s high frequency of excitation ensures that the assembly operation occurs well below the threshold for fully-developed cavitation, enabling the assembly process to proceed under the influence of fluid forces that have relatively high spatial uniformity. The megasonic excitation also provides the necessary diversity of fluid forces, including both the out-of-plane primary forces that drive the selective removal process and the in-plane secondary (acoustic streaming) forces that drive component circulation. The assembly fluid’s water content enables the hydrophobic interactions that drive assembly. The transducer provides the acoustic excitation that enables the selectivity in the assembly process. The intensity of the acoustic excitation is controlled via the transducer’s input voltage.

The TMPTA microcylinders are prepared for assembly by suspending them in the water/ethanol mixture and dispersing them in the fluid by shaking the mixture on a vortex mixing tool (Vortex-2 Genie, by Scientific Industries, Bohemia, NY, USA) for five minutes. The microcylinders are used immediately after dispersion to avoid settling. Since the TMPTA micro-components are naturally hydrophobic, no chemical surface functionalization is required. About 2 mL of the assembly fluid medium is pipetted into the assembly beaker, and the template is placed face-up in this beaker. A sufficient volume (500 μL, at a density of 10,000 components/mL) of each of the two component solutions (one containing 26 μm nominal diameter components with 50 μm length and one containing 26 μm nominal diameter components with 150 μm length) is simultaneously added to the assembly beaker. This provides a modest oversupply of components, with more than five times as many microcylinders as assembly sites on the template.

After the small beaker is capped, the transducer’s power is turned on and the experiment is allowed to proceed undisturbed for five minutes. Five minutes was chosen by increasing the assembly time in 30 second increments until the assembly yield had robustly saturated. Assembly results were examined at transducer voltages of 40, 45, 50, 55, and 60 V to identify the operating point that optimizes the assembly yield. An optimal transducer voltage of 45 V was identified and is used in subsequent experiments. Above this voltage, assembly is suppressed because the removal effects are too strong [18,19]; below this voltage, assembly is suppressed because the component circulation is too weak for the components to effectively sample the assembly sites [19,20]. At the optimal voltage, the acoustic excitation of the fluid mixture promotes the selective removal of components from incorrectly matched sites on the template while simultaneously ensuring adequate circulation of components over the template surface in the fluid. The template is then removed at an angle from the upper surface of the assembly mixture, placed on an external flat surface, allowed to air-dry for about a minute, and examined under an optical microscope. The mass density of the components is greater than the mass density of the assembly liquid, ensuring that the number density of components at the upper surface of the assembly mixture is very small. Because few components occupy the small volume of liquid that remains on the upper surface of the chip after its removal from the top of the assembly mixture, the risk of increasing the number of components on the surface or in the sites due to capillary forces during air-drying is low, and the effect on assembly results is insignificant [19].

A key metric for the assembly success is the assembly yield, which is the ratio of the number of sites that are filled with components to the total number of sites. After the assembly yield is quantified, components are detached from the assembly sites with ultrasonic excitation (kHz range) and recaptured to enable reuse of the components and template. Conventional ultrasonic excitation in the kHz range is used because it creates cavitation, thereby dislodging all matched and unmatched components from the assembly sites.

## 4. Results and Discussion

**Figure 5:**
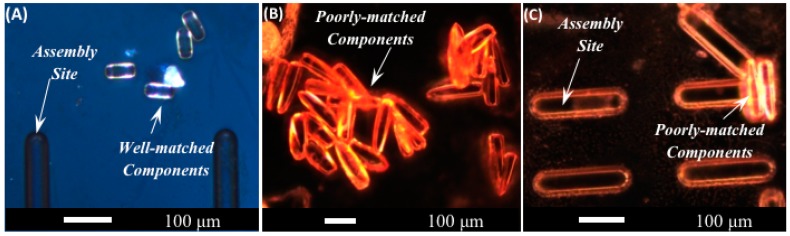
Optical micrographs of portions of the assembly template before assembly, showing two sets of anisotropic assembly components lying on (but not assembled into) the assembly template. (**A**) shows the shorter components that are well-matched to the template geometry, with 26 μm diameter and 50 μm length. (**B**,**C**) show the longer components that are poorly-matched to the template geometry. The poorly-matched components have a length of 150 μm and taper from a maximum diameter of 26 μm to a visibly narrower point at the opposite end.

Figure 5 shows optical micrographs of the two sets of TMPTA components used for testing shape and size selectivity of assembly.

**Figure 6:**
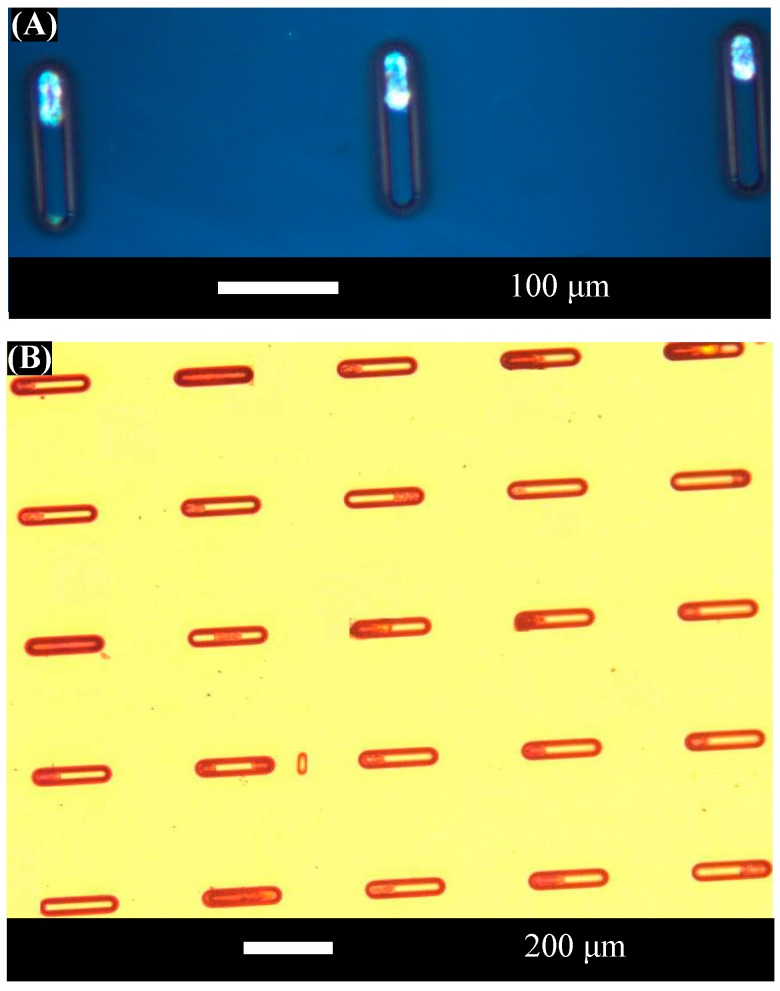
Optical micrographs showing portions of the assembly template after assembly of cylindrical components. (**A**) shows a higher magnification view of assembly into wells 120 μm in length. (**B**) shows a lower magnification view of assembly into a large number of wells 200 μm in length. Variations in well appearance after assembly reflect where components are located in the wells and how many components are assembled in each well.

These images are taken prior to the assembly process, so the components are not located in assembly wells. The taper of the longer components is visible in the figure. Figure 6 shows optical micrographs of 26 μm diameter, 50 μm long microcylinders after successful assembly at 45 V into the assembly wells described previously.

These results indicate that the wells are sufficiently well-matched to promote assembly of the shorter components. In contrast, assembly of the nominally 26 μm diameter, 150 μm long tapered microcylinders into assembly wells was unsuccessful. One may ask whether the failure of the longer, tapered components to assemble into the sites reflects their failure to enter the sites or the successful operation of the selective removal mechanism. From the point of view of diameter matching, the tapered components are well-matched to the dimensions of the assembly sites at their widest ends; however, the tapered components are narrower than the assembly sites along the rest of their lengths. Previous research using *in situ* inspection with an immersed lens [20] demonstrated that spherical components that are smaller than an assembly site can enter the site and are subsequently removed by the fluid excitations. Other prior research [18] examined the assembly rates of too-small spherical components in larger assembly sites, finding that too-small components will assemble into larger sites if the fluid excitation is too weak to remove them. Since the physics of rolling surface contact and fluid excitations are similar in the cylindrical system and in the spherical system, the results of [18,20] support the same conclusion for cylinders. Namely, the fact that only the well-matched cylindrical components are retained in the assembly sites while the poorly-matched, tapered components are removed supports the successful operation of the selective removal process.

The shape-matching model also predicts that the tapered components will be removed from the assembly sites. The tapered components have a maximum radius (at one end) of 13 μm and a minimum radius (at the other end) of 5 μm. Idealizing the taper as a linear transition from the maximum radius end to the minimum radius end yields an average radius of curvature of 9 μm for the tapered components. Evaluating the shape-matching model from above with this average radius and the tapered components’ experimentally-implemented length of 150 μm yields a retention moment to removal moment ratio of 0.21. The fact that the ratio is almost five times less than the critical value of one indicates that the tapered components should be removed in the selective removal process, as is experimentally observed. Although the average radius approximation is not exact, the large (factor of five) reduction of the ratio below its critical value of one is robust against the partially compensating errors due to overestimation of the radius at the narrower end and the underestimation of the radius at the wider end.

The selective removal of poorly-matched components was successful as predicted by the shape-matching model. In addition, it was successful despite the potential for plastic deformation in hydrogel materials, as was predicted by the deformation model. Only diameter matching is demonstrated in these experiments since the 50 μm long, well-matched components are shorter in length than the assembly sites. Figure 6 shows that most of the components assemble at the ends of the assembly sites. This is consistent with the fact that contact area between the components and the template is maximized (and interfacial energy is minimized) when the cylinders, which have rounded end caps, are positioned at the ends of the assembly sites instead of in the middle of an assembly site.

The results show that the cylinders’ long axes successfully align with the long axes of the assembly sites in the final assembled structures. The rapid success in aligning the anisotropic components with their anisotropic assembly sites is attributed to the physics of the assembly system. The components travel along the surface under the influence of the acoustic streaming flow, which is a steady flow that arises secondary to the primary oscillatory effects of the acoustic excitation [30]. The fluid’s velocity gradient near the boundary produces a net fluid torque as well as a net fluid force on the components. The net torque ensures that rolling comprises one part of the components’ motion along the surface, while the net force ensures net forward motion and prevents tapered components from purely rolling in circles. As is shown in [20], components must overcome an energy barrier arising from a reduced contact area at the edge of an assembly site when they first approach it. This energy barrier slows the progression of the component into the well, as demonstrated via immersed lens studies; full details are available in [20]. For the anisotropic components used here, when a component is not initially parallel to the assembly site, one end of the component reaches the edge of the assembly site first. The energy barrier at the edge of the well slows the progression of the first end of the component. In fact, the resistance from the edge of the site is expected to be stronger for a cylinder than it is for a sphere, since a cylinder makes contact with the surface about a line instead of about a point as in the case of a sphere. Together, the distributed fluid force and the point force applied to one end of the component by the assembly site create both a couple and a net force. The couple rotates the component in the plane so that the opposite end of the component “catches up” to the end of the component that reached the site first. The couple therefore rotates the component into alignment with the assembly site. Once the component is aligned with the assembly site, it is free to roll into the site. In this way, the energy landscape not only controls the selectivity of the assembly process, but also drives the alignment of the components with the assembly sites.

The rotating alignment process poses a greater challenge for longer components than it does for shorter components. To lowest order, the location along an assembly site at which a component first makes contact with the assembly site is random. Because the rotation occurs about that point of first contact, it is possible that when the component is parallel to the site, its length may not be completely contained within the length of the assembly site. If this occurs, the component will be unable to enter the assembly site. The chances of this occurring are larger when the length of the component is commensurate with the length of the site (e.g., for the 150 μm long tapered components and the 200 μm long assembly sites) than when the components are much shorter than the assembly sites. For example, there is a 25% chance that a 50 μm long component will first contact a 200 μm long assembly site at a point that will preclude assembly after rotation, as compared with a 75% chance that a 150 μm long component will first contact the same site at a location that will ultimately prevent assembly due to lengthwise misalignment. This effect may be expected to reduce the assembly rate of the longer, tapered components, but it will not prevent their assembly entirely. The fact that the assembly of tapered components is almost entirely unsuccessful therefore reflects the success of the selective assembly process as well as the challenges of initial assembly for longer components.

The five-minute time required to achieve robust assembly of the microcylinders (*i.e.*, assembly in which further increases in assembly time do not significantly increase the assembly yield) is the same as the five-minute assembly time required to achieve robust assembly of microspheres in [21,23]. The fact that five minutes also achieves successful assembly for the present anisotropic components indicates that the need to achieve alignment does not significantly extend the required assembly time as compared with the case of isotropic components.

The assembly yield was quantified by calculating the ratio of the number of holes of each size that are occupied by components to the total number of holes of that size. The sites with components inside them (one or multiple, irrespective of location inside site) comprise successful assembly. Assembly yields for the shorter, well-matched components are high, as expected from the results shown in Figure 6. The experiments recorded here correspond to a local assembly yield of 96% for the 50 μm long, 26 μm diameter microcylinders when the acoustic transducer is driven at 45 V; local yield is measured over a smaller 5 × 5 array of 25 assembly sites. Because of the low ratio of components to assembly sites, local regions were selected for analysis from near the center of the template. Near the center of the template, maximum component circulation minimizes the effects of a low component to site ratio, aiding initial component assembly. The 5 × 5 arrays were chosen from the central area of the template, which is the region of the substrate with the highest concentration of assembled parts.

In contrast, the measured assembly yield for the longer, tapered components is 2% (as measured over 120 assembly sites). The wildly divergent measured yields between well-matched and tapered components demonstrate the key role of shape matching in directed assembly of anisotropic components using the present approach. The high local assembly yield for well-matched components also indicates that, despite the low average component density in the assembly fluid, the process works efficiently in regions of the fluid where flow patterns ensure that sufficiently many components are available. The results also suggest that global assembly yield may be improved by using a larger number of components, improving the circulation of components within the assembly fluid, or moving the template within the fluid during the assembly process to ensure that all parts of the template are exposed to high component density portions of the assembly bath.

The variation in local yield among multiple measurements for nominally identical assembly conditions was less than 6%. The locally high assembly yield recorded for well-matched assembly components confirms that the selective removal process of the present assembly technique works well in this geometry and material system, in agreement with the model predictions that shape matching will be effective for microcylinders and that the system will not be deformed into the plastic regime, ensuring that the assembly process is not hindered by energy dissipation. Together with the model predictions, these results establish that assembly of anisotropic objects on rigid substrates can be predicted with tools that are similar to those used for isotropic systems, and that the present selective assembly approach is a valuable tool for rapid, precise, and flexible shape-matched structuring of small-scale anisotropic systems.

## 5. Conclusions

Successful selective assembly of anisotropic, cylindrical components out of a mixed group of matched (cylindrical) and unmatched (tapered) components was presented. Only the components that were well matched in shape and size, with diameters that were uniformly similar to the diameters of the assembly sites, were retained inside the sites. High local assembly yield was obtained using the present assembly process. Components were observed to occupy the ends of the assembly sites to maximize contact area, consistent with the predictions of selective removal theory [18,19]. The global assembly yield was moderate due to the overall low component density in the system. However, overall yield may potentially be increased by raising the component density or engineering better circulation of the existing components among the assembly sites.

The theoretical model presented here offers tools to predict the extent to which shape-matching enables selective assembly of cylinders and tools to predict the circumstances under which a given set of materials and geometries will be successfully assembled with the present selective assembly process. The model is validated by the successful selective assembly of well-matched, plastically-deformable hydrogel micro-components into silicon-based substrates, and by the successful rejection of poorly-matched components of the same hydrogel material.

## Figures and Tables

**Table 1:** Predicted retention moment, removal moment, and moment ratio for cylindrical components of various radii.

Radius of Cylindrical Component, *R_c_* (μm)	Retention Moment, *T_ret_* (Nm)	Removal Moment, *T_rem_* (Nm)	Ratio of Moments, T*_ret_/T_rem_*
6.0	2.82 × 10^−14^	4.67 × 10^−13^	0.06
7.0	8.37 × 10^−14^	7.43 × 10^−13^	0.11
10.0	5.86 × 10^−13^	2.17 × 10^−12^	0.27
12.0	1.64 × 10^−12^	3.74 × 10^−12^	0.44
12.5	1.95 × 10^−12^	4.23 × 10^−12^	0.46
13.0	3.05 × 10^−12^	4.76 × 10^−12^	0.64
13.5	6.33 × 10^−12^	5.33 × 10^−12^	1.19

**Table 2:** Approach distance calculated on a flat surface and comparison with critical approach values. Calculations assume a cylindrical microcomponent with diameter of 26 μm and a contact length of 50 μm and an applied contact load of 3.4 × 10^−5^ N.

Component Material	Approach Distance, α (m)	Critical Approach Distance, α*_c_* (m)	α/α*_c_*
Polystyrene (PS)	1.28 × 10^−9^	9.02 × 10^−9^	1.42 × 10^−1^
PDMS	3.15 × 10^−^^6^	5.56 × 10^−^^3^	5.66 × 10^−^^4^
PMMA	1.18 × 10^−^^9^	1.15 × 10^−^^7^	1.03 × 10^−^^2^
Melamine	3.70 × 10^−^^10^	2.90 × 10^−^^8^	1.28 × 10^−^^2^
Mammalian cell	3.01 × 10^−^^6^	5.15 × 10^−^^3^	5.85 × 10^−^^4^
Teflon	6.10 × 10^−^^9^	2.93 × 10^−^^7^	2.08 × 10^−^^2^
Polypropylene	1.64 × 10^−^^9^	2.11 × 10^−^^7^	7.78 × 10^−^^3^
Poly(ethylene glycol) (PEG)	3.60 × 10^−^^7^	2.47 × 10^−^^5^	1.46 × 10^−^^2^
Poly(lactic acid) (PLA)	4.94 × 10^−^^10^	5.13 × 10^−^^8^	9.64 × 10^−^^3^
Poly(glycolic acid) (PGA)	1.16 × 10^−^^9^	1.28 × 10^−^^7^	9.03 × 10^−^^3^
Poly(urethane acrylate) (PUA)	2.69 × 10^−^^9^	1.24 × 10^−^^7^	2.18 × 10^−^^2^

**Table 3:** Approach distance calculated inside a hemicylindrical assembly site and comparison with critical approach values. Calculations assume a mean well diameter of 27 μm, a cylindrical microcomponent with diameter of 26 μm and contact length of 50 μm, and an applied contact load of 3.3 × 10*^−^*^3^ N.

Component Material	Approach Distance, α (m)	Critical Approach Distance, α*_c_* (m)	α/α*_c_*
Polystyrene (PS)	7.42 × 10^−8^	8.21 × 10^−8^	9.04 × 10^−1^
PDMS	5.14 × 10^−^^5^	4.93 × 10^−^^1^	1.04 × 10^−^^4^
PMMA	6.84 × 10^−^^8^	9.20 × 10^−^^7^	7.43 × 10^−^^2^
Melamine	2.25 × 10^−^^8^	2.51 × 10^−^^7^	8.96 × 10^−^^2^
Mammalian cell	5.06 × 10^−^^5^	4.72 × 10^−^^1^	1.07 × 10^−^^4^
Teflon	3.24 × 10^−^^7^	2.18 × 10^−^^6^	1.49 × 10^−^^1^
Polypropylene	9.37 × 10^−^^8^	1.62 × 10^−^^6^	5.80 × 10^−^^2^
Poly(ethylene glycol) (PEG)	1.24 × 10^−^^5^	5.49 × 10^−^^5^	2.26 × 10^−^^1^
Poly(lactic acid) (PLA)	2.97 × 10^−^^8^	4.31 × 10^−^^7^	6.90 × 10^−^^2^
Poly(glycolic acid) (PGA)	6.73 × 10^−^^8^	1.02 × 10^−^^6^	6.59 × 10^−^^2^
Poly(urethane acrylate) (PUA)	1.50 × 10^−^^7^	9.85 × 10^−^^7^	1.52 × 10^−^^1^
